# Examining the Impact of a Novel Blood Glucose Monitor With Color Range Indicator on Decision-Making in Patients With Type 1 and Type 2 Diabetes and its Association With Patient Numeracy Level

**DOI:** 10.2196/diabetes.8299

**Published:** 2017-10-02

**Authors:** Mike Grady, Laurence Barry Katz, Christine Simone Strunk, Hilary Cameron, Brian Leonard Levy

**Affiliations:** 1 LifeScan Scotland Inverness United Kingdom; 2 LifeScan Inc Wayne, PA United States

**Keywords:** color range indicator (ColorSure™ Technology), glucose ranges, blood glucose monitor, self-monitoring of blood glucose, numeracy

## Abstract

**Background:**

Many patients struggle to interpret and respond appropriately to the numerical blood glucose results displayed on their meter, with many regularly taking no action or self-care adjustment for out-of-range results. We recently reported that a glucose meter that provides automatic onscreen information using a color range indicator (ColorSure Technology) improved the ability of patients to categorize their blood glucose results.

**Objective:**

The objective of this study was to examine how ColorSure Technology (or color) affected patient decision making on blood glucose results and how patient numeracy levels influenced such decisions.

**Methods:**

We invited 103 subjects (56 with type 2 diabetes and 47 with type 1 diabetes) to a face-to-face in-clinic visit in a diabetes care center and showed them glucose results with or without color via interactive computer or paper logbook exercises. Before participating in these exercises, subjects completed surveys on numeracy and their understanding of blood glucose information.

**Results:**

Subjects preferentially acted on high glucose results shown with color (55%, 57/103) compared to results without color (45%, 46/103; *P*=.001). When shown identical pairs of results, subjects preferentially acted on results shown with color (62%, 64/103) compared to results without color (16%, 16/103) (*P*<.001). Subjects more accurately identified days of the week in which results were low, in range, or high when reviewing logbooks with color (83%, 85/103) than without color (68%, 70/103; *P*=.012). Subjects with lower numeracy were more likely to consider taking action for high glucose results shown with color (59%, 18/31) than without color (41%, 13/31) and preferentially would take action on results shown with color (71%, 22/31) compared to results without color (16%, 5/31).

**Conclusions:**

Insulin- and noninsulin-using subjects were each more inclined to act when glucose results were shown with color, and associating glucose results with color was viewed as particularly beneficial by subjects with lower numeracy.

## Introduction

Self-monitoring of blood glucose (SMBG) remains a cornerstone of diabetes management. However, poor education on how to meaningfully interpret the numbers displayed, together with a lack of understanding about adequate responses to blood glucose (BG) levels, can diminish the value of self-monitoring [1]. Appropriate education addressing SMBG interpretation and response to “out-of-range” BG values has been identified as a prerequisite to the value of SMBG [2]. In people with type 1 diabetes (T1D), underutilization of SMBG and absence of guided clinical decision making have recently been identified as key contributors to poor glycemic control [3]. Furthermore, Cavanagh et al [4] described how low diabetes-related numeracy skills are associated with fewer self-management behaviors. Poor numeracy has also been associated with suboptimal glycemic outcomes in people with both type 2 diabetes (T2D) [5] and T1D [6].

We previously reported that although nearly all patients with T2D agreed they would take action for BG results under 70 mg/dl, 51% of these subjects stated they would not take action for any level of high BG result [7]. This is consistent with a study of 207 people with T2D that investigated perceptions of high BG results where only 28% of patients considered results >235 mg/dl as high, with a further 10% viewing only >290mg/dl as high [8]. This demonstrates a recurring tolerance (or lack of awareness) of high BG levels in people with T2D. We previously reported that a variety of blood glucose meters (BGM) using color range indicators improved the ability of patients with both T1D and T2D to interpret and classify BG readings into low, in range, or high glucose ranges [9]. In the current study, we investigated how color might influence decision making in people with T1D and T2D in terms of propensity to take action after low or high BG results. In addition, we explored the impact of numeracy on decision making and subject preference for results in color.

## Methods

This single visit, open label study was conducted at a National Health Service (NHS) clinic in the United Kingdom (Highland Diabetes Institute [HDI], Scotland) and was approved by the relevant ethics committee. Subjects provided written informed consent before initiation of the study. Subjects were identified via the NHS patient electronic database, based on entrance criteria, and were invited to attend the clinic by a clinic research nurse. Inclusion criteria included at least 16 years of age, an ability to read and understand English, a diagnosis of diabetes (T1D or T2D) for at least 3 months, and self-reported SMBG of at least 1 time per day. The only exclusion criterion was conflict of interest, that is, subject was not or had previously not been employed with LifeScan Scotland, or had a family member or association with LifeScan Scotland. Subjects provided demographic, medical history, and current diabetes practice information to the study facilitator. In addition, the subject’s most recent laboratory A1c result was obtained from the NHS database. All subjects received appropriate compensation for time and travel to the clinic site. HDI is a stand-alone facility in Inverness, Scotland, adjacent to a general hospital (Raigmore Hospital). HDI cares for more complex or difficult to manage people with diabetes who usually have been referred from general practice. Therefore, HDI typically has a higher proportion of more intensively managed patients (eg, multiple daily insulin injections or using pumps) than might be encountered if recruiting via general practice. This is reflected in our study demographics.

### ColorSure Technology Feature

**Figure 1:**
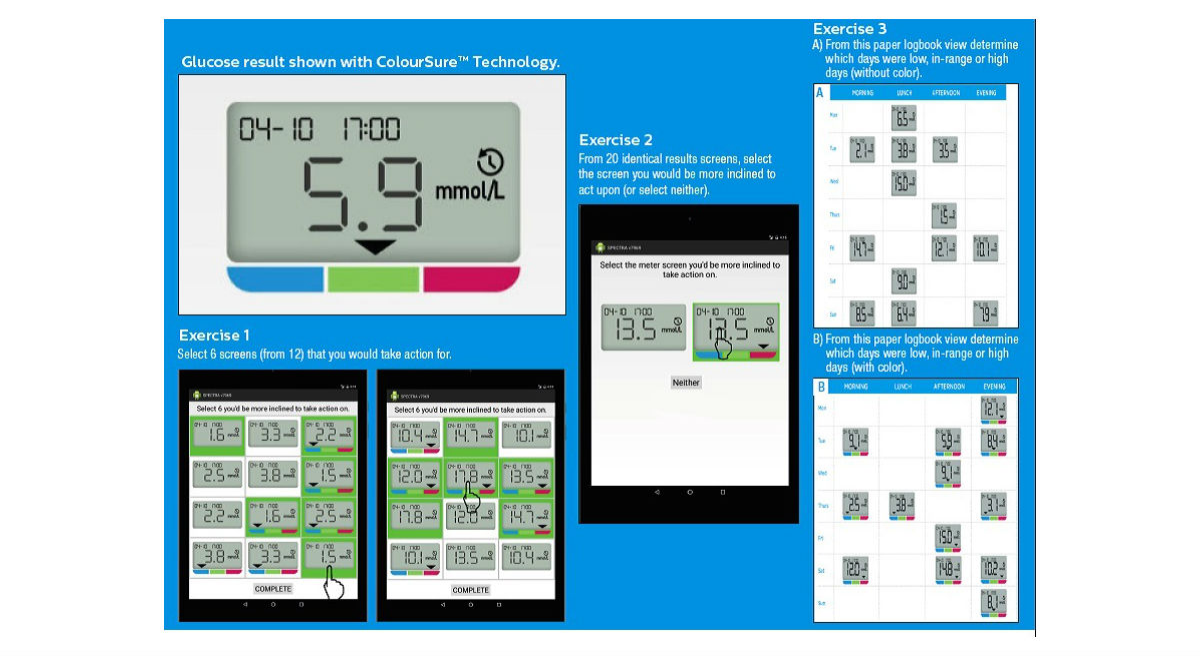
ColorSure technology and subject exercises.

Subjects interacted with study materials using a computer or by handling paper-based study materials that described blood glucose information with the support of ColorSure Technology (CST) (LifeScan). CST describes a way of presenting blood glucose data to a patient (on a glucose meter or app) in association with color (blue, green, or red) to denote low, in range, or high glucose results, respectively (Figure 1). CST is used in OT Verio, OT Select Plus, and OT Verio Flex blood glucose meters (LifeScan). This feature automatically indicates whether a glucose result displayed on the screen is low (blue), in range (green), or high (red) (Figure 1). The determination of a low, in range, or high message depends on the glucose range set in the meter by the patient or health care professional (HCP). Low (<70mg/dl), in range (70-180mg/dl), and high (>180mg/dl) default limits are provided preset in the meter and were used in this study.

### Assessing Glucose Data (With or Without) Color

Three different exercises were undertaken by each subject. Exercises #1 and #2 were facilitated using a tablet personal computer (PC) enabling each subject to view different meter screens and provide feedback by clicking directly on the tablet screen to record their response. Exercise #3 involved reviewing two different paper logbooks containing typical glucose results screens.

#### Exercise #1: Reviewing Low or High Glucose Results With or Without Color

Each subject was shown a single panel view of 12 low (<70 mg/dl) or high (>180 mg/dl) glucose result screens on the tablet PC (Figure 1). This single panel view consisted of 6 identical pairs of low and 6 identical pairs of high results shown with or without color. Subjects were asked to click directly on 6 of the possible 12 low and high result screens on which they would be more inclined to take action.

#### Exercise #2: Reviewing Glucose Pairs With or Without Color

A series of 20 identical pairs of 20 different glucose results were shown one at a time on the tablet PC screen in random order (Figure 1). Each pair of numerically identical results screens was shown on the tablet PC simultaneously side-by-side: one result shown with color and the other result shown without color. Each subject was asked to select the results screen on which they would be more inclined to take action. Subjects could also select “neither,” meaning no preference for results with or without color.

#### Exercise #3: Reviewing Blood Glucose Logbooks With or Without Color

Each subject reviewed two different one-page paper logbooks displaying a representative week of results. One logbook displayed 13 results with color, and the other logbook displayed 13 results without color (Figure 1). The two logbooks had glucose results placed on different days and were numerically only marginally different from each other. Within each logbook, 3 of the 7 days had specific results that exhibited a low, in range, or high blood glucose pattern. The facilitator first presented the logbook without color and then presented the logbook with color to each subject and asked them to identify which days of the week results were typically running low, in range, or high. Subjects were also asked preference questions after reviewing each form of logbook.

### Timing of Exercises

In Exercises 1 and 2, both black/white and color visuals were presented in the same exercise simultaneously; therefore, no time advantage (or disadvantage) was implicit in the choice that was made by the subject when expressing a preference. Therefore, there was no rationale for measuring the time taken to conduct the exercises.

For Exercise 3 (using paper logbooks), a time limit of 2 minutes for interpretation of the black/white logbook and a further 2-minute time limit for interpretation of the color logbook was enforced.

#### Subjective Numeracy Scale Evaluation

All subjects took part in a subjective numeracy assessment using a validated subjective numeracy scale [10,11]. Subjects read 8 statements and chose from 6 potential responses (scored from 1-6, with 6 defining highest self-reported confidence or ability in terms of numeracy) that most represented themselves. A total subjective numeracy score (between 8-48) was determined for each subject. To facilitate the interpretation of numeracy scores, we classified results into 5 categories (8-16, 17-24, 25-32, 33-40, and 41-48) to facilitate understanding of lowest to highest subjective numeracy across subjects.

#### Subject Surveys

After all study procedures were completed, subjects completed surveys to investigate their knowledge of glucose ranges and explore how subjects interpret and react to low or high results. Finally, subjects expressed their perception of the value of the color feature with respect to managing their diabetes.

#### Statistical Analyses

Continuous demographic variables were described as median and range or mean and standard deviation (SD). Categorical demographic variables were described as percentages within categories. Test score changes were calculated as the percentage change from baseline. The null hypothesis “H_0_: Pre-score=post-score” was tested using a paired *t* test with significance level of alpha=.05. Correlations with A1c and other variables were assessed using the Pearson correlation coefficient and were deemed significant with *P*<.05. Minitab 16.1.1 and SPSS 21.0 were used for all analyses.

**Table 1:** Baseline patient demographics and medical history.

		All subjects (N=103)	T1D (n=47)	T2D (n=56)
**Gender, n (%)**				
	Male	47 (45.6)	20 (42.6)	27 (48.2)
	Female	56 (54.4)	27 (57.4)	29 (51.8)
Age (years), mean (SD)		61.6 (14.1)	55.4 (15.4)	66.7 (10.6)
**Years conducting SMBG**				
	Mean (SD) 18.0 (9.6)	23.9 (9.4)	13.0 (6.6)	
	Median (range)	16.8 (1.8-39.8)	26.8 (2.8-39.8)	12.8 (1.8-31.8)
**Frequency of SMBG, n (%)**				
	>5 times/day	20 (19.4)	20 (42.6)	‒
	3-5 times/day	46 (44.7)	17 (36.2)	29 (51.8)
	1-2 times/day	32 (31.1)	9 (19.1)	23 (41.1)
	<1 time/day	5 (4.9)	1 (2.1)	4 (7.1)
**Therapy, n (%)**				
	Insulin pump	8 (7.8)	8 (17.0)	17 (30.4)
	Insulin injections	47 (45.6)	30 (63.8)	‒
	Insulin injections and oral antidiabetes drugs	40 (38.8)	9 (19.1)	31 (55.4)
	Oral antidiabetes drugs only	8 (7.8)	‒	8 (14.3)
**HbA1c (%)**				
	Mean (SD)	8.3 (1.4)	8.3 (1.3)	8.3 (1.4)
	Median (range)	7.9 (5.4-12.4)	7.9 (5.4-12.4)	8.0 (5.6-12.0)

## Results

### Subjects

Baseline characteristics of subjects are shown in Table 1. In total, 47 subjects with T1D and 56 subjects with T2D participated. The majority (86%, 48/56) of the subjects with T2D used some form of insulin. All subjects were experienced SMBG users who performed BG tests relatively frequently (64%, 66/103), performing at least 3 tests per day.

#### Assessing Blood Glucose Data (With or Without) Color

**Figure 2:**
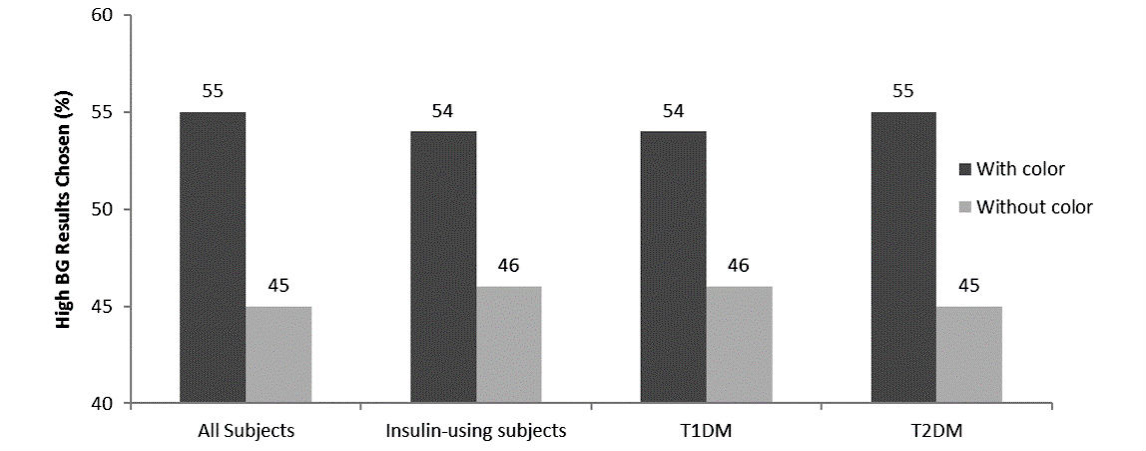
Preference for subjects to act on high BG results with and without color. All subjects (N=103); insulin-using subjects (n=94); T1DM (n=47); T2DM (n=56).

In Exercise #1, there was no significant difference across the 103 subjects in terms of choosing to preferentially act whether the low result was shown with color (52%, 54/103) or without color (48%, 49/103). This outcome was not influenced by whether the subject had T1D or T2D. However, there was a significant difference across the 103 subjects in terms of choosing to preferentially act when identical high results were shown with color (55%, 57/103) compared to without color (45%, 46/103) (*P*=.001) (Figure 2). This preference for results with color was also observed across the 94 insulin-using subjects who chose to preferentially act when identical high results were shown with color (54%, 51/94) compared to without color (46%, 43/94) (*P*=.012) (Figure 2).

**Figure 3:**
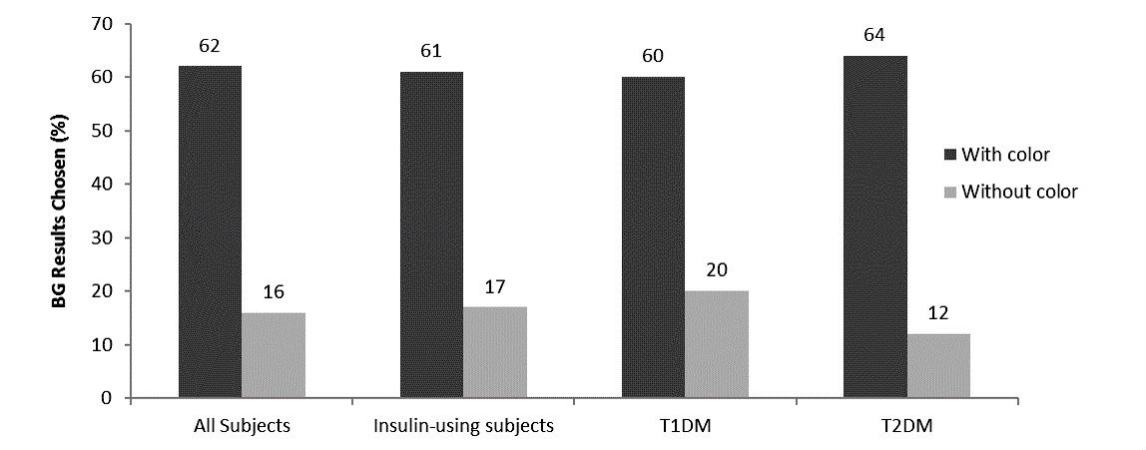
Preference of subjects to view individual BG results with or without color on a meter screen. The remaining subjects expressed no preference between color and no color. All subjects (N=103); insulin-using subjects (n=94); T1DM (n=47); T2DM (n=56).

Similarly, in Exercise #2 there was a significant difference across the 103 subjects in the percentage of subjects choosing to preferentially act on a result shown with color (62%, 64/103) compared to the same numeric result shown without color (16%, 16/103) (*P*<.001) (Figure 3). The remaining subjects expressed no preference between color and no color. This preference for results with color was also observed across the 94 insulin-using subjects (61%, 57/94 vs 17%, 16/94; *P*<.001). In addition, this response preference for results with color was seen in the 47 subjects with T1D (color 61%, 29/47; without color 20%, 9/47) and the 56 subjects with T2D (color 64%, 36/56; without color 12%, 7/56), respectively (Figure 3).

#### Reviewing Blood Glucose Logbooks With or Without Color

**Figure 4:**
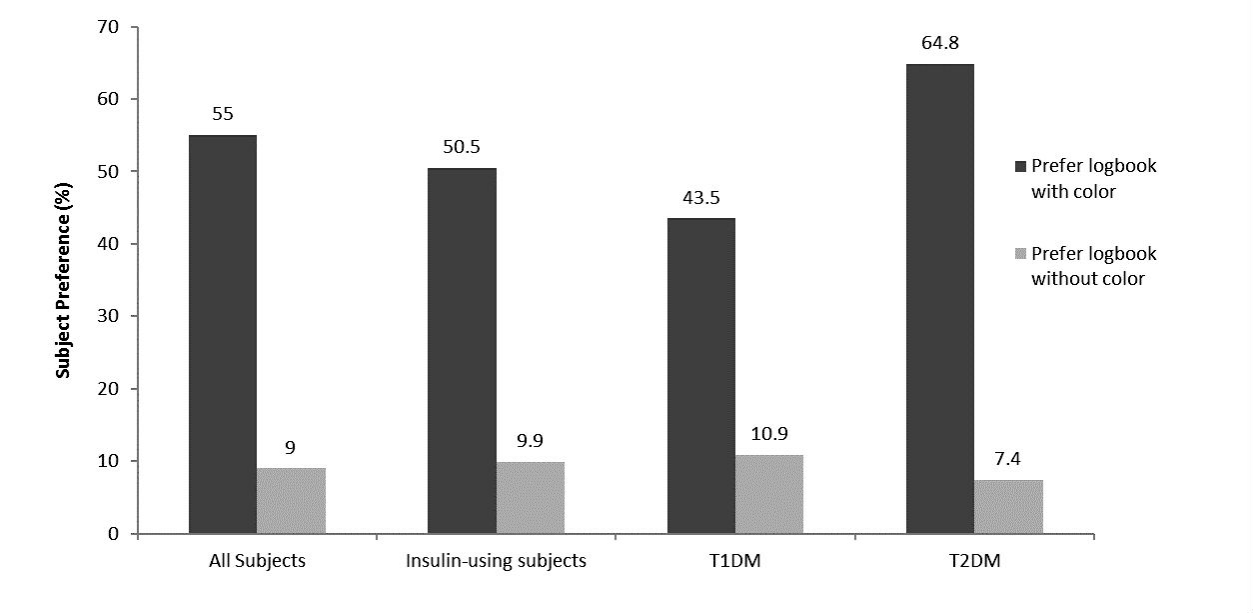
Preferences for subjects to review BG data in a logbook with or without color. The remaining subjects expressed no preference between color and no color. All subjects (N=103); insulin-using subjects (n=94); T1DM (n=47); T2DM (n=56).

In Exercise #3, more subjects correctly identified the 3 days when results were low, in range, or high when reviewing logbooks with color (83%, 85/103) compared to without color (68%, 70/103) (*P=*.012) This improvement was also evident across the 91 insulin-using subjects (82%, 75/91) compared to insulin users reviewing logbooks without color (66%, 60/91) (*P*=.01). Over half (55%, 57/103) of subjects responded that a logbook displaying results with color was easier to review compared to only 9% (9/103) who preferred a logbook without color. The remaining subjects expressed no preference between color and no color. Preference for reviewing glycemic trends using a logbook with color was also more pronounced in subjects using insulin, subjects with T1D, and subjects with T2D (Figure 4).

#### Subject Numeracy and Associations With Baseline Measures

**Figure 5:**
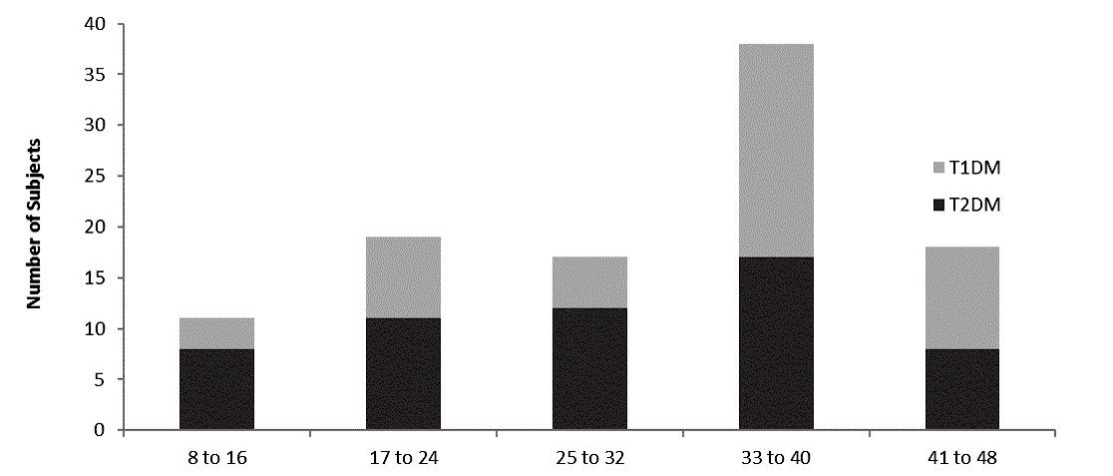
Numeracy level across subjects: Subjective Numeracy Scale scores in 103 subjects, T1DM (n=47) and T2DM (n=56). The 8-question scale has 6 items per question with a maximum score of 48 representing highest subjective numeracy evaluation and a minimum score of 8 representing lowest subjective numeracy evaluation. Numbers represent the number of subjects scoring in the range shown.

Median subjective numeracy score was 34 (minimum possible score, 8; maximum possible score, 48) across all 103 subjects with a range of 8-48 (11-48 T1D; 8-48 T2D) (Figure 5). There was no correlation between numeracy and either A1c or SMBG frequency across all subjects or within subjects with either T1D or T2D.

#### Subject Numeracy and Associations With Color

**Figure 6:**
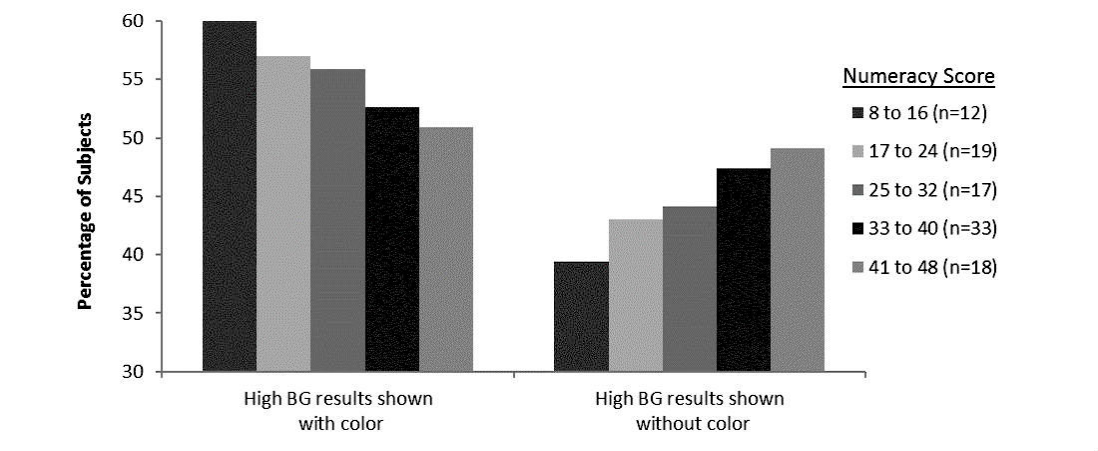
Numeracy associations between subjects choosing high BG results when 6 identical BG results were shown with or without color.

In Exercise #1, subjects with lower numeracy levels (8-24) were more likely to say they would take action for high results shown with color (59%, 18/31) than without color (41%, 13/31). As numeracy level increased, subjects became noticeably less reliant on color to identify high results (Figure 6). For example, at the highest subjective numeracy level (score of 41-48), an equivalent number of subjects chose to take action for high results regardless of whether values were shown with or without color.

**Figure 7:**
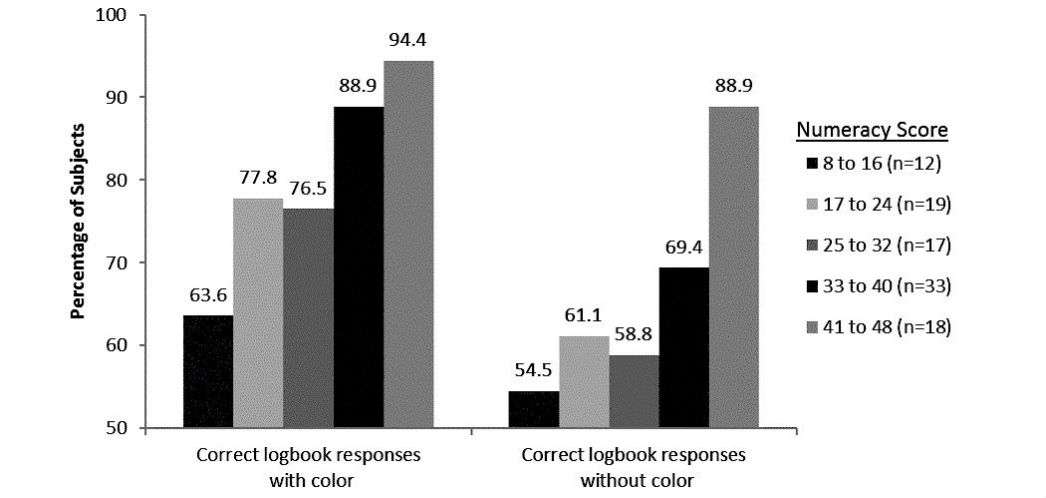
Association between numeracy and ability of subjects to correctly identify low, in range, or high days when results were shown with or without color in logbook format.

During Exercise #2, when reviewing identical pairs of glucose results shown with or without color, subjects with lower numeracy (8-24) preferentially selected action for results shown with color (71%, 22/31) compared to those without color (16%, 5/31). This preference to take action based on color declined as numeracy improved but was still evident even in subjects with the highest numeracy level where 53% (10/18) would preferentially take action for results shown with color compared to 16% (3/18) without color. During logbook Exercise #3, as numeracy improved, individual subjects were more successful at correctly identifying days of the week containing low, in range, or high glucose results (Figure 7). This trend was evident whether results were displayed with or without color. However, at every numeracy level, subjects were always more successful at identifying days of the week containing low, in range, or high results from logbook reviews with color (Figure 7).

#### Subject Perceptions of ColorSure Technology Feature

More than two-thirds (68%, 70/103) of subjects agreed or strongly agreed that showing a result with color (CST) makes it simpler to know when to act compared to a meter without color and that color could help them understand when they need to take action. In subjects with T2D, subjects agreed that color could help them improve awareness of when blood glucose is low (71%, 40/56) or high (66%, 37/56) and that showing a result with color could make it clearer when to take action (68%, 38/56). In T1D and T2D subjects with lower numeracy (equivalent to 51% [53/103] of all subjects based on a median numeracy score of <34), these subjects agreed that color could help them improve awareness of hypoglycemic (66%, 35/53) or hyperglycemic (70%, 37/53) results and that color would motivate them to stay in range (66%, 35/53) and feel more confident managing diabetes between scheduled HCP visits compared to a meter without color (64%, 34/53) (Table 2).

**Table 2:** Subject responses to survey statements. Favorable responses are defined as a response of “strongly agree” or “agree” on a 5-point scale (5=strongly agree, 4=agree, 3=neither agree nor disagree, 2=disagree, and 1=strongly disagree). Neutral responses are a score of 3. Nonfavorable responses are a score of 1 or 2. All favorable response rates are statistically significant (*P*<.05).

		Nonfavorable response	Neutral response	Favorable response
**All subjects (N=103)**				
	ColorSure could help me understand when I need to take action along with the BG number	12%	25%	63%
	ColorSure provides extra awareness about what BG results mean^a^	16%	24%	60%
	Showing a result with ColorSure makes it simpler to know when to act^b^	16%	16%	68%
	ColorSure improves the testing experience compared to using a meter without any color	3%	34%	63%
**Subjects with T2DM (n=56)**				
	ColorSure could help me improve my awareness of when my blood glucose is low	16%	13%	71%
	ColorSure could help me improve my awareness of when my blood glucose is high	16%	18%	66%
	ColorSure could give me more confidence to understand my results^a^	16%	21%	63%
	ColorSure could make it clearer when I need to take action compared to a number only	16%	16%	68%
	Showing a result with ColorSure makes you more inclined to act compared to seeing your result without color	20%	14%	64%
**Subjects with low numeracy^c^(n=53; 21 T1DM, 32 T2DM)**				
	ColorSure could help me improve my awareness of when my blood glucose is low	11%	23%	66%
	ColorSure could help me improve my awareness of when my blood glucose is high	11%	19%	70%
	Using a meter with ColorSure could help me take the right steps to manage my blood glucose	10%	26%	64%
	Using a meter with ColorSure could help me be more confident when I need to take action along with the BG number	9%	23%	68%
	ColorSure would make me feel more confident managing my diabetes between scheduled HCP visits^a^	11%	25%	64%
	ColorSure could motivate me to stay in-range between HCP visits^a^	11%	23%	66%
	ColorSure could help me recognize signs and avoid trouble spots between HCP visits^a^	9%	25%	66%

^a^Compared to a meter without color.

^b^Compared to a result without color.

^c^Subjective Numeracy Scores less than the median of all study participants.

## Discussion

### Principal Findings

Building on our previous studies that showed that color enables patients with T1D and T2D to improve their ability to interpret blood glucose readings [7,9], we sought to demonstrate that color has the potential to improve the likelihood that patients will act on results. This is especially important given that many patients stop taking action over time, especially when they have high results, and often avoid taking any action whatsoever [8,12]. We demonstrated that color can positively influence the intention of subjects to act on glucose values and that this effect was particularly evident in subjects with lower numeracy. CST automatically highlights when individual results are within accepted glycemic ranges (low, in range, or high) and provides a simple association with color to reinforce how patients should interpret their results and facilitate appropriate action.

Our study demonstrated that color helped patients recognize when they should consider taking action in response to certain results, especially high results. The fact that color had less of an influence on whether subjects would consider taking action on low results may reflect the importance placed by HCPs on educating and reminding patients how to identify and react to hypoglycemia. In contrast, subjects responded more readily in terms of inclination to take action when high results were presented to them with color compared to high results shown without color. The strong subject preferences for intention to take action when viewing results displayed with color may point to a deficiency in education regarding what is a high value for that individual and what action could be taken in the moment or what prospective therapy or lifestyle changes could be adopted to minimize future high results. Interestingly, subjects with both T1D and T2D had similar preference for color when asked to consider a series of results on which they would preferentially intend to act. Our results imply that the immediate reassurance provided by color appeals equally to both groups even if anecdotally patients with T1D feel that color is less instructive to them given their higher testing frequency and greater familiarity reviewing blood glucose information.

A recent study in 7320 people with T2D not using insulin [13] found that in 1 of 6 people who practiced SMBG, neither the patient nor physician used any SMBG results to make treatment adjustments. These patients reported either diabetes was not a high priority for them or their HCP did not teach them how to adjust diet/medicines based on SMBG results. The reaction of the T2D population in our study (86%, 48/56 insulin users) who were performing SMBG at least 3 times a day is perhaps more similar to T1D subjects in terms of SMBG awareness or interpretation. This may explain why the reactions of subjects with T2D in our study to color were similar to the reactions of subjects with T1D.

It is well known that both patients and HCPs struggle to decipher glycemic pattern information from logbooks [14], which are often unclear or inaccurate [15]. We have found that a logbook presenting blood glucose results with color may overcome immediate barriers to deciphering trends within a logbook for patients with both T1D and T2D.

Strong trends were noted with respect to subject numeracy and reviewing SMBG data with color. In particular, subjects with lower numeracy were far more likely to say they would act on high results when presented with color. Consistent with this finding, Cavanagh et al [4] found 26% of 398 patients surveyed could not identify values within a target range of 60-120 mg/dl, and this declined further to 33% in those with the lowest numeracy. In contrast, patients with the highest numeracy were able to identify results within the target range 88% of the time. Additionally, in our study, about 2 out of 3 subjects with below-median numeracy felt that color would make them feel more confident managing diabetes between visits and could also help them recognize signs and avoid trouble between HCP visits.

The preference for viewing and acting on results with color may also have benefits in terms of reinforcing appropriate decision making over time. For example, there exists a disparity in the perception of patients and HCPs on how well patients can interpret SMBG data. A recent study noted that 38% of physicians perceived that nurses “always” assessed patients’ ability and knowledge with respect to SMBG and when to take action, whereas only 14% of the patients felt they were “always” taught how to perform SMBG or given information regarding treatment based on SMBG results [16]. Therefore, HCPs may be overestimating how effectively their patients can interpret SMBG data.

### Limitations

HDI cares for more complex, intensively managing patients (eg, multiple daily insulin injections or using pumps) than might be encountered in general practice and this is reflected in our study demographics; 86% (48/56) of our T2D subjects were taking some form of insulin and 52% (29/56) performed SMBG ≥3 times per day, much higher than people with T2D in the general local population. Despite our intensively managing study population having familiarity with SMBG data and access to expert care from diabetes specialist nurses, it was encouraging that participants still appeared to benefit from, and exhibit strong preferences for, color-coded information. It is possible that the value of color insights may be even stronger in a more generalized T2D population who typically perform SMBG less frequently and may be less able to interpret numerical glucose data.

We acknowledge that there are relatively small numbers of subjects within the lowest numeracy level (8-16), which limits robustness of the data. However, there are clear overall trends associating changes in numeracy with subject performance or preference for color. The tablet PC system used to enable subjects to experience a wide range of glucose results (with or without) color is admittedly a simulation for results on an actual glucose meter, but it allowed subjects to quickly and easily visualize sequential meter screen images or meter screens in parallel and respond in the moment. These are the kind of assessments we expect patients to make after each glucose test at home (often multiple times per day). This concentrated experience, viewing a series of glucose values, is an efficient way to obtain an estimation of each subjects’ ability and perceptions concerning interpreting data with or without color.

The paper logbook exercises were not randomized; subjects always completed the black/white logbook assessment first followed by the color logbook assessment. Familiarity with the format of the materials and process may have helped some subjects. Justification for this order was that performing the color logbook first would provide additional education on what represented a low, in range, or high result, which could have influenced or improved interpretation of the standard logbook. It is also worth highlighting that subjects were given a time limit of 2 minutes to assess each logbook in turn and with the exception of 1 subject (a retired T2D male testing the minimum of 1 time per day) who provided 5 of the required 6 selections in time, no other subjects skipped any selections for any logbook in the required timeframe. On this basis, it is clear that even if we assume that there was a learning curve in witnessing the black/white logbook first, the fact that everyone completed the exercises in such a short time suggests it was not a clinically meaningful advantage.

### Conclusion

Both insulin and noninsulin-using subjects may benefit from color to support interpretation of blood glucose results displayed either on a meter or in a logbook. Our study suggests strong preferences for viewing results with color and that subjects may be more inclined to act, particularly on hyperglycemic results, when presented with results in color. Displaying glucose results with color improves interpretation of SMBG results and can assist and encourage subjects to act on SMBG data, which may enable them to follow their HCP recommendations more closely between scheduled consultations.

## Abbreviations

A1c: hemoglobin A1c
BG: blood glucose
BGM: blood glucose monitor
CRI: color range indicator
HCP: health care professional
SD: standard deviation
SMBG: self-monitoring of blood glucose
T1D: type 1 diabetes
T2D: type 2 diabetes
